# Cannabidiol Protects Dopaminergic Neurons in Mesencephalic Cultures against the Complex I Inhibitor Rotenone Via Modulation of Heme Oxygenase Activity and Bilirubin

**DOI:** 10.3390/antiox9020135

**Published:** 2020-02-04

**Authors:** Johanna Catharina Duvigneau, Alice Trovato, Andrea Müllebner, Ingrid Miller, Christopher Krewenka, Kristina Krenn, Wilhelm Zich, Rudolf Moldzio

**Affiliations:** Institute of Medical Biochemistry, University of Veterinary Medicine Vienna, Veterinaerplatz 1, 1210 Vienna, Austria; Catharina.Duvigneau@vetmeduni.ac.at (J.C.D.); alicce@hotmail.de (A.T.); andrea.muellebner@vetmeduni.ac.at (A.M.); Ingrid.Miller@vetmeduni.ac.at (I.M.); christopher.krewenka@vetmeduni.ac.at (C.K.); kristinakrenn@gmx.at (K.K.); zichwilli@aon.at (W.Z.)

**Keywords:** phytocannabinoids, heme oxygenase, biliverdin reductase, bilirubin, rotenone, neuroprotection

## Abstract

Phytocannabinoids protect neurons against stressful conditions, possibly via the heme oxygenase (HO) system. In cultures of primary mesencephalic neurons and neuroblastoma cells, we determined the capability of cannabidiol (CBD) and tetrahydrocannabinol (THC) to counteract effects elicited by complex I-inhibitor rotenone by analyzing neuron viability, morphology, gene expression of IL6, CHOP, XBP1, HO-1 (stress response), and HO-2, and in vitro HO activity. Incubation with rotenone led to a moderate stress response but massive degeneration of dopaminergic neurons (DN) in primary mesencephalic cultures. Both phytocannabinoids inhibited in-vitro HO activity, with CBD being more potent. Inhibition of the enzyme reaction was not restricted to neuronal cells and occurred in a non-competitive manner. Although CBD itself decreased viability of the DNs (from 100 to 78%), in combination with rotenone, it moderately increased survival from 28.6 to 42.4%. When the heme degradation product bilirubin (BR) was added together with CBD, rotenone-mediated degeneration of DN was completely abolished, resulting in approximately the number of DN determined with CBD alone (77.5%). Using N18TG2 neuroblastoma cells, we explored the neuroprotective mechanism underlying the combined action of CBD and BR. CBD triggered the expression of HO-1 and other cell stress markers. Co-treatment with rotenone resulted in the super-induction of HO-1 and an increased in-vitro HO-activity. Co-application of BR completely mitigated the rotenone-induced stress response. Our findings indicate that CBD induces HO-1 and increases the cellular capacity to convert heme when stressful conditions are met. Our data further suggest that CBD via HO may confer full protection against (oxidative) stress when endogenous levels of BR are sufficiently high.

## 1. Introduction

### 1.1. Phytocannabinoids as Protectants in Neurodegenerative Diseases

Phytocannabinoids (CBs) from *Cannabis sativa* are discussed to provide neuroprotection against chronic neurodegenerative disorders like multiple sclerosis, Huntington’s disease, Parkinson’s disease (PD), Alzheimer’s disease (AD), and amyotrophic lateral sclerosis [1,2]. For many neurodegenerative diseases, impairment of mitochondrial function resulting in enhanced oxidative stress has been shown [3,4,5,6]. In several in-vitro models of mitochondrial dysfunction, especially oxidative stress models relevant to PD, neuroprotective effects of CBs have been demonstrated. Tetrahydrocannabinol (THC) and cannabidiol (CBD) are protective for dopaminergic neurons damaged by oxidative stress induced by the inhibition of complex I of the electron transport chain and cell degeneration produced by glutamate [7,8,9]. Glutamate-induced oxidative stress is believed to occur through inhibition of cysteine/glutamate antiporter, resulting in depletion of glutathione (GSH) levels and accumulation of reactive oxygen species (ROS) [10,11]. Therefore, the antioxidative properties of CBs may be of high relevance for neuroprotection. Clinical data from PD patients support this assumption: nigrostriatal lesions are associated with an increase in CB1 receptors in the basal ganglia [12].

While these effects are at least partly explained by CBs acting as ligands for the endocannabinoid system, in particular via the interaction with cannabinoid receptor 1 (CB1), CBs may also directly exert antioxidant activities [8,13]. Likewise, CBs may directly modulate the activities of enzymes involved in oxidative processes. Inhibition of the enzyme reaction of several isoforms of the cytochrome P450 system by CBs has been shown [14]. Therefore, it is well possible that some of the protective mechanisms against oxidative stress exerted by CBs do not require the involvement of cannabinoid receptors. Since heme oxygenase (HO) and the products generated by HO activity constitute an effective cellular antioxidative defense system (see below), we hypothesized that CBs might exert neuroprotection by engaging the HO system.

### 1.2. Role of HO and the Biliverdin Reductase (BVR) System in Neurodegeneration and Neuroprotection

HO degrades heme to ferrous iron, carbon monoxide (CO) and biliverdin (BV). BVR converts BV to the stable product bilirubin (BR). Activities of HO and BVR are high in nervous tissues, and heme degradation products have been shown to play important roles in neuronal function [15,16,17,18] and neuroprotection [19,20,21,22]. Oxidative and inflammatory challenges upregulate the inducible form of heme oxygenase (HO-1), also known as heat shock protein 32 (HSP32), and BVR [23,24]. Therefore, HO-1 is believed to confer protection, especially in conditions of increased oxidative stress [24,25,26,27,28]. Also, the constitutive HO isoform (HO-2), predominantly active in neuronal tissues, contributes to the function and survival of neurons via the generated products CO and BV/BR [29]. Increased HO-1 and BVR levels were monitored in patients with AD [30,31,32], and these patients display increased levels of BR in the cerebrospinal fluid [33]. Additionally, in the early stages of PD, increased levels of BR were found [34]. Although BR in higher concentrations is a known neurotoxin [35,36], it has been found to exert distinct neuroprotective effects [37,38,39]. Protection is explained by the antioxidant capabilities of the BV/BR cycle, assumed to protect the membrane/water interface [40,41,42]. Although its relevance as an endogenous antioxidant system is controversially discussed [43], BR decreases the stress-induced inflammatory response [19,44].

### 1.3. Phytocannabinoids and Interaction With the HO and BVR System

Only a few reports suggest an association of CBs with the heme degradation pathway. It has been shown that HO-1 induction modulates the cannabinoid receptor 2 (CB2) activity and that vice versa, anti-inflammatory effects of CB2 engagement require up-regulation of HO-1 protein [45,46]. Little is known whether cannabinoids are capable of modulating the activity of the HO enzyme. However, CBs are able to modulate the expression of HO-1. CBD treatment of glial cells resulted in an increased expression of genes belonging to the axis, which is activated in response to oxidative stress, in particular, the gene controlled by the transcription factor Nrf2, which also triggers expression of HO-1 [47]. Smooth muscle cells show an increased expression of HO-1 in response to CBD treatment [48]. Therefore, upon CBD treatment, increased HO activity could be expected. In an in-vivo model using mice subjected to a long-term treatment with a CB1 agonist, hepatic tissue displayed an increased heme degradation capacity [49]. Whether changed HO activity is involved in neuroprotection exerted by CBs, has not been elucidated yet. It is conceivable that one of the mechanisms underlying the protective effects of CBs is to trigger antioxidative defense via increased HO activity.

### 1.4. Experimental Models to Study Neurodegeneration and the Mechanisms Underlying Cytoprotection of CBs

In order to address the role of oxidative stress in neurodegenerative diseases, substances such as 1-methyl-4-phenylpyridinium (MPP^+^) or rotenone, both inhibitors of mitochondrial complex I, are used in both cellular and animal models [7,50]. MPP^+^ affects only dopaminergic neurons since it enters cells via dopamine transporters, while rotenone is taken up by all cells. In sensitive cells, rotenone increases the formation of ROS, especially superoxide radicals, and decreases ATP levels, leading to a severe imbalance in energy metabolism. Betarbet et al. showed that rotenone administration leads to the formation of Lewy bodies that are characteristic neuronal aggregates in PD patients [51]. 

A widely used model for inducing mitochondrial impairment related to neurodegeneration is the primary mesencephalic cell culture. In the substantia nigra, dopaminergic neurons are much more affected by oxidative challenges than other neurons as their basal level of ROS is higher due to dopamine metabolism [52]. Due to the higher vulnerability of dopaminergic neurons, these cells degenerate in PD, leading to motor and subsequent cognitive loss of function. 

In our previous study, we reported that the CBs THC and CBD mediated neuroprotection in MPP^+^-treated primary mesencephalic cultures. However, the effective concentration of 10 µM CBD, in contrast to the other two CBs, exerted neurotoxic effects by itself [7]. It is not clear yet which mechanisms underlie these seemingly opposite effects. The above-mentioned induction of HO-1 by CBD indicates that CBD itself acts as a stressor and may increase oxidative stress. 

We hypothesize that CBD, similar to other stressors, may protect neurons via this increased stress response and thereby confer tolerance to additional oxidant insults. This phenomenon is called “preparation for oxidative stress” [53], and explains the mechanism underlying the protective conditioning effects. CBD has been shown to act as neuroprotective agent against stressful conditions in several in-vivo and in-vitro models [7,8,9,54,55,56,57], possessing antioxidant and anti-inflammatory activity [58]. We hypothesized that the described protective mechanism exerted by CBD involves an antioxidative stress response exerted via the HO system. Given the important role of heme degradation products, in particular, BR for neuroprotection [21,39,55], we further hypothesize that CBD mediates neuroprotection involving the upregulation of HO activity.

## 2. Materials and Methods 

### 2.1. Cell Culture

J774A.1 cells were purchased from ATCC (Manassas, VI, USA) and N18 TG2 cells from the DSMZ (Braunschweig, Germany), Pregnant OF1/SPF mice were purchased from the Institute for Laboratory Zoology and Veterinary Genetics, Himberg (Austria). All cells were cultured in an incubator at 37 °C, with 5% CO_2_, and 100% humidity.

#### 2.1.1. Primary Mesencephalic Cultures 

The study was discussed and approved by the ethical and animal welfare committee of the University of Veterinary Medicine Vienna (ETK-04/09/2015) in accordance with the GSP guidelines and the Austrian Act on Animal Experiments, which corresponds to the guidelines of the European Union Council (2010/63EU) for the use of laboratory animals. On gestation day 14, pregnant mice were sacrificed by cervical dislocation after deep CO_2_ anaesthetization. The ventral mesencephalon of the embryos were excised, meninges were removed, and tissues were mechanically fragmented. Tissue pellets were carefully triturated in serum-containing medium (DMEM supplemented with 8.7 mM HEPES, 9.7 mM glucose, 1.7 mM glutamine, and 8.7% fetal bovine serum; all from Sigma-Aldrich, Taufkirchen, Germany) using fire-polished Pasteur pipettes. Dissociated cells were resuspended in serum containing medium. Mesencephalic cells were plated on poly-D-lysine-coated 48-well plates (density 750,000 cells/mL, 340 µL per well). The culture medium was exchanged on the 1st day in vitro (DIV) and 3rd DIV. On 5th DIV, half of the medium was replaced by serum-free medium containing DMEM medium supplemented with 9.4 mM HEPES, 10.4 mM glucose, 1.9 mM glutamine, and 1.4% B27 supplement (Thermo Fisher Scientific, Vienna, Austria). From the 6th DIV on, serum-free B27 medium was used and changed every 2nd day. On the 12th DIV, solutions of bilirubin, CBD, THC (all 10 µM), and rotenone (80 nM, a concentration previously tested to damage cells, but which can be counteracted by CBs, data not shown) were prepared from dimethyl sulfoxide stocks (DMSO, Merck, Darmstadt, Austria) in serum free medium and administered to the cells for 48 h. The DMSO concentration in the culture medium did not exceed 0.01%. 

#### 2.1.2. Neuronal N18TG2 Adherent Cell Cultures and J774A.1 Macrophages Grown in Suspension

N18TG2 neuroblastoma cells were kept in DMEM supplemented with 9.7% fetal bovine serum, 1.94 mM glutamine, and 1.94 mM sodium pyruvate (Sigma-Aldrich, Vienna, Austria), and split when reaching 80% confluence. Treatment of neuroblastoma cells for viability testing was performed in 96-well plates. Cells were collected by centrifugation for 10 min at 178× *g* and resuspended in colourless DMEM (supplemented with 1.94 mM glutamine, 1.94 mM sodium pyruvate, and 2% of B27 supplement) in a final concentration of 3 × 10^5^ cells/mL. All treatment conditions (see above) were made in duplicates. 

J774A.1 was grown as suspension using DMEM supplemented with 10% fetal bovine serum, 4 mM glutamine, and 1 mM sodium pyruvate (Sigma-Aldrich, Vienna, Austria). Cells were collected by centrifugation for 5 min at 300× *g*; the cell pellet was homogenized in 100 µL HO assay buffer (100 mM potassium phosphate buffer containing 1 mM EDTA, pH 7.4) per 10^6^ cells, snap-frozen in liquid nitrogen and stored at −80 °C until being used.

#### 2.1.3. Determination of Cell Number and Viability

All compounds used to elucidate the potential interaction of CBs with the HO system investigated in this study were checked for their potential cytotoxicity for both, neuroblastoma N18TG2 cells, and primary mesencephalic cultures using different test systems. 

(A) Tyrosine Hydroxylase (TH) Staining for Determination of Surviving Neurons in Primary Mesencephalic Cultures 

On the 14th DIV, cultures were fixed in 4% Histochoice (Sigma-Aldrich, Vienna, Austria) for 10 min at 4 °C, washed in Dulbecco´s phosphate buffered saline (DPBS, Thermo Fisher Scientific, Vienna, Austria), followed by permeabilization with 0.4% Triton X-100 for 30 min in DPBS (pH 7.2, Roche, Vienna, Austria) at room temperature (RT). Between the following steps, cultures were washed twice with PBS for 3 min at RT. Thereafter, cells were incubated with anti-tyrosine hydroxylase antibody (1:1000 in 5% horse serum containing DPBS, Szabo-Scandic, Vienna, Austria) at 4 °C overnight. The biotinylated secondary antibody was incubated for 90 min at RT, followed by incubation with avidin-biotin-horseradish peroxidase complex according to the manufacturer’s protocol (ABC Kit, Vector Laboratories, Petersborough, UK). Immunoreaction was visualized using 1.4 mM diaminobenzidine (DAB, Sigma-Aldrich, Taufkirchen, Germany) solution containing 3.3 mM hydrogen peroxide. Finally, cells were covered with Kaiser’s glycerol gelatin (Merck, Vienna, Austria). The total number of tyrosine-hydroxylase immunoreactive (THir) cells was evaluated in 18–22 fields/well using a Nikon inverted microscope at 100× magnification. 

(B) Resazurin Reduction Assay

In order to measure the overall metabolic activity, 4 µM resazurin (Sigma-Aldrich, Vienna, Austria) was added to the culture medium. The time-dependent reduction was assessed after 0 and 2 h by measuring the absorbance at 570 and 600 nm (Tecan Sunrise, Tecan, Männedorf, Switzerland). The cell cultures were kept at 37 °C and 5% CO_2_ between measurements. The reduction of resazurin was calculated as [(O_2_ × A_1_ − O_1_ × A_2_)/(R_1_ × N_2_ − R_2_ × N_1_)] × 100 with the absorbances: A_1_ = test probes at 570 nm; A_2_ = test probes at 600 nm; N_1_ = negative control (no cells) at 570 nm; N_2_ = negative control (no cells) at 600 nm, and the molar extinction coefficients (E): O_1_ = oxidized resazurin at 570 nm (80.586 L·mol^−1^·cm^−1^); O_2_ = oxidized resazurin at 600 nm (117.216 L·mol^−1^·cm^−1^); R_1_ = reduced resazurin at 570 nm (155.677 L·mol^−1^·cm^−1^); R_2_ = reduced resazurin at 600 nm (14.652 L·mol^−1^·cm^−1^); The reduction of resazurin of the vehicle control was set to 100% of cell viability. 

(C) Crystal Violet Assay

Cell viability was evaluated using the crystal violet assay cells. For staining, the medium was discarded, and the adherent cells fixed with pure ethanol (96%, −20 °C, Sigma-Aldrich, Taufkirchen, Germany) for 15 min at −20 °C. Then, crystal violet (0.1% in 10% ethanol) was incubated for 15 min in the dark. The dyeing solution was removed, and the wells washed three times with dH_2_O. The dishes were kept at RT and in the dark to dry for at least 24 h. The staining was then solubilized in 300 μL acetic acid (10%) and measured at 590 nm wavelength in the plate reader. 

### 2.2. Determination of HO Activity

#### 2.2.1. Preparation of Samples

After treatment for 48 h in a 12 well plate, cells were washed with PBS and the plates were layered with 300 µL HO assay buffer (100 mM potassium phosphate buffer containing 1 mM EDTA, pH 7.4) per well and frozen at −80 °C. Prior to use, the frozen cells were resuspended. Liver from adult male Sprague Dawley rats weighing 300–400 g was homogenized in a 1:10 (*w:v*) ratio in Tris-buffer containing 300 mM sucrose, 20 mM Tris, and 2 mM EDTA at pH 7.4 using a Potter-Elvehjem with polytetrafluoroethylene (PTFE) pestle on ice. The homogenates were distributed as 150 µL portions and snap-frozen into liquid nitrogen until being used. Occasionally, cell homogenates were treated by sonication, and the homogenate was then used for the determination of the HO-activity and protein content using Coomassie Brilliant Blue (Bradford assay).

#### 2.2.2. HO Enzyme Assay

We used a biochemical assay described recently [59] with some modifications. Cell homogenate (50–100 µg protein) was added to a reaction mixture containing 500 nmol nicotinamide adenine dinucleotide phosphate (NADPH) and 20 nmol of hemin in HO assay buffer (final volume 150 µL). The mixture was incubated in darkness for 30 min at 37 °C under constant agitation and afterward transferred on ice to stop the reaction. For enhancing BR extraction 50 µL caffeine-solution (0.25 mol/L caffeine, 0.52 mol/L sodium benzoate, 0.9 mol/L sodium acetate) and 50 µL saturated KCl were added. The formed BR was extracted into benzene (4× the assay volume) by vigorously mixing (3 × 30 s) the samples. Phase separation was enabled by centrifugation (400× *g*) for 15 min at RT. The organic extract was harvested and the BR concentration determined using a double beam spectrophotometer (U 3000, Hitachi, Vienna, Austria). The samples were measured in duplicates using the following instrument settings using the inbuilt software UV solutions 2.2: 400–600 nm; slit 2 nm, scan speed: 120 nm/min; photomultiplier tube: autogain; high resolution; 2 repeats. BR concentration in extracts was determined using a standard calibration curve. Standards were generated by adding known amounts of BR to assay buffer followed by extraction into benzene, as described above. The protein concentration of tissue homogenate was determined using Coomassie Brilliant Blue (Bradford assay). Enzyme activities were expressed as nmol BR formed per mg protein in 30 min. The values were corrected for the amount of BR determined in negative controls (incubation without substrate and NADPH), and for the samples where BR was used as stimulant additionally in the presence of 200 nmol of the HO inhibitor zinc (II), protoporphyrin IX. Further, it was verified that the activity of BVR sufficiently exceeds that of HO (between five to 10 times) to ensure that all BV formed by the HO reaction was rapidly converted to BR (data not shown). Additionally, we verified that BR is not subjected to degradation by enzyme activities or oxidation processes mediated by tissue homogenates (please refer to the Supplementary Figure S1). Since material deriving from cultured cells was limited, we determined HO activity for each data point only by two consecutive experiments, which corresponds to two biological replicates. Therefore, statistical analyses were not performed.

#### 2.2.3. HO Enzyme Kinetics

Determination of the inhibitory potential of CBs on the HO enzyme reaction was determined by endpoint analyses (as described in Section 2.2.2). Prior to testing the mode of inhibition, the inhibitory concentration 50 (IC_50_) of CBs was determined using dilution series of CBD and THC, respectively, of which each dilution was added to the assay and compared to the vehicle control assembled in parallel. The obtained dose response curves were used to calculate the respective IC_50_ by polynomial regression. After verification that the extrapolated IC_50_ is indeed leading to an inhibition of 50% in the homogenates of J774A.1 cells, the dilution series of the substrate heme was prepared, covering a broad concentration range (0.02–800 µM final hemin concentration). The two series were prepared from two stock solutions of hemin in DMSO (20 and 10 mM, respectively) by multiple consecutive 1:3 dilution steps. All the dilutions were used for endpoint analyses of the HO activity, performed in duplicates. Due to the high quantity of samples, the data were generated in several experiments using both hemin dilution series with and without the added CBD at IC_50_. Data were pooled to generate the dose-response curves. To generate the Lineweaver-Burk plot, the BR specific optical density (OD, equivalent to the amount of BR) was displayed against the input hemin concentration in the dynamic range (0.65–16 µM) in a double reciprocal fashion.

### 2.3. Gene Expression Analysis

#### 2.3.1. Preparation of Samples

N18TG2 cells were grown in the presence of CBD, THC, BR, rotenone, and vehicle alone or in combination using 6- or (as indicated in Section 2.1.2) 12-well plates. After 16 h, the cell culture medium was removed, and cells were lysed in 1 mL of TriReagent^®^ (Molecular Research Center Inc., Cincinnati, OH, USA) per well.

#### 2.3.2. RNA Extraction and cDNA Synthesis

RNA extraction was performed according to the manufacturer’s protocol. The amount and purity of the extracted RNA were determined with an Eppendorf BioPhotometer plusUV/VIS (Eppendorf, Wesseling-Berzdorf, Germany), using absorption at 260 nm and the 260/280 nm ratio, respectively. One µg of total RNA was reversely transcribed to cDNA using anchored oligo-dT-primers (3.5 μmol/L final concentration) and reverse transcriptase (Superscript™ II RNAse H; 200 U/reaction; Invitrogen; Carlsbad, CA, USA). An internal standard (IS) was used as a reference for the quantification of qPCR, which was generated by pooling equal aliquots from each cDNA.

#### 2.3.3. Quantification of Target mRNA Expression by qPCR

Analysis of gene expression was performed by means of qPCR (for additional information see Tables S1 and S2 and Figure S2). Primer pairs for the analysis of mRNA expression of HO-1, HO-2, interleukin (IL) 6, C/EBP homologous protein (CHOP), glucose-regulated protein (GRP) 78 and X-box protein (Xbp) 1, as well as the internal reference gene Cyclophilin A (Cyclo), were previously described [60,61,62,63,64]. QPCR was performed using a CFX96™ (Bio-Rad, Hercules, CA, USA). Each reaction contained SYBR^®^ green I as reporter (0.5×, Sigma Aldrich, Vienna, Austria), iTaq™ polymerase™ (0.625 U/reaction; BioRad, Hercules, CA, USA), the primers (250 nmol/L each, Invitrogen; Carlsbad, CA, USA) at a final concentration of 200 μmol/L dNTP (each). In all the reactions, 3 mmol/L MgCl_2_ were used, except for CHOP, which was amplified with 2 mmol/L supplemented to the provided reaction buffer at a final volume of 12 μL. All reactions were performed in duplicates. Corresponding, randomly assigned no-reverse-transcriptase (NRT) controls (15% of all samples investigated), the no-template-control (NTC), as well as the IS were included in each measurement. Data analysis was performed using the inbuilt software CFX manager (Version 2.0, Bio-Rad, Hercules, CA, USA) in the linear regression mode. Target gene expression was calculated against the IS and normalized by ΔCq values obtained for the internal reference gene, as previously described [65]. The ΔΔCq values obtained from the replicates were averaged and calculated as 2^–ΔΔCq^ values and were expressed in fold changes relative to the vehicle control.

### 2.4. Data Processing and Statistics

Data obtained for the HO activity were displayed as means ± standard deviation (SD). Statistical calculation was not done, since for each condition, two independent experiments were performed only due to the limitation of the material. All other experiments were repeated at least three times, and therefore, data were expressed as means ± standard error of the mean (SEM). For qPCR data, we used a one-way ANOVA with Bonferroni correction using SPSS/PASW 17.0 (IBM, New York, NY, USA) to calculate significant differences (*p* < 0.05). For experiments evaluating the survival of dopaminergic neurons and for the metabolic activity in N18TG2 cells, the Kruskal-Wallis test was used, followed by the pairwise comparison with non-parametric Mann-Whitney U-test. Differences were considered statistically significant when *p* < 0.05. Interaction between stress induction (rotenone vs. control) and treatment (vehicle, CBD, THC) and enhancer (vehicle vs. BR), indicative for a mechanistic association between these factors, was investigated using univariate analysis of variance with SPSS/PASW 17.0. An alpha level of α < 0.05 was used as the criterion for statistical significance. To show statistical differences, we used one symbol when the criteria of *p* < 0.05 were fulfilled without further scaling for different significance levels. In line with the suggestion of Amrhein et al. [66], the exact *p* values for each statistical test are shown in the supplementary tables (Tables S3–S6).

## 3. Results

To obtain insight into the potential association of the neuroprotection exerted by CBs with the HO system, we studied the ability of the CBs to modulate the HO activity in primary neurons present in cultured mesencephalic preparations and the neuronal cell line N18TG2. We investigated the protective potential of CBs against cell stress and neuronal cell death induced by the well-known complex I inhibitor rotenone.

### 3.1. Modulation of HO Activity by CBs

**Figure 1:**
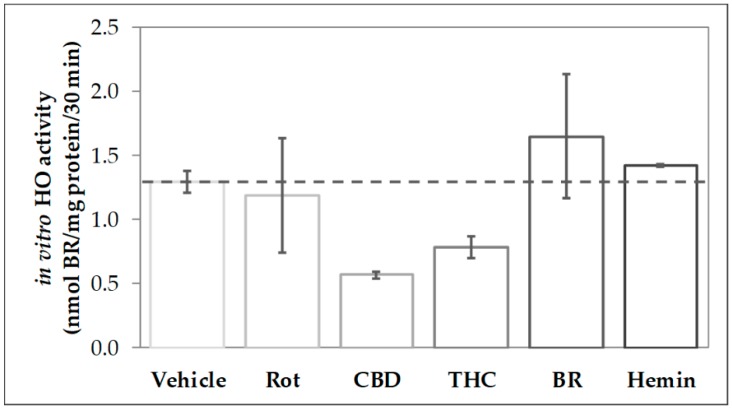
Phytocannabinoid treatment reduces the heme-converting capacity in primary mesencephalic cultures. Mesencephalic cultures were obtained and cultured, as is described in the Material and Methods. Cells were incubated with vehicle dimethyl sulfoxide (DMSO), rotenone (Rot, 80 nM), cannabidiol (CBD; 10 µM), tetrahydrocannabinol (THC; 10 µM), bilirubin (BR; 10 µM), or hemin (10 µM) for 48 h. Heme oxygenase (HO) activity was determined as described in Material and Methods. Each data point represents the mean ± SD from two experiments (*n* = 2), which were measured in duplicates. Therefore, statistical analyses were not performed.

In order to find out whether CBs modulate the activity of HO, we analyzed the capability to convert heme in homogenates of primary mesencephalic cells subjected to a 48-h treatment with the stressor rotenone, the CBs CBD and THC as well as the HO product BR and the HO substrate heme (Figure 1). We found that cells treated with CBD for 48 h displayed only 44% of the heme degrading capacity (in-vitro HO activity) compared to control cells (vehicle). Also, THC-treatment reduced the heme converting capacity, albeit to a lesser extent than CBD. In contrast, HO activity was not affected by treating the cells with rotenone (80 nM), BR (10 µM), or heme (10 µM). 

**Figure 2:**
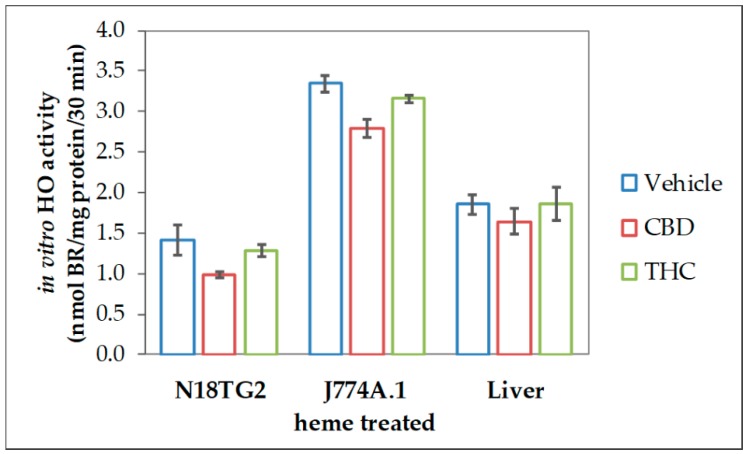
Cannabidiol exerts inhibitory effects on heme oxygenase activity in variable tissues. We used homogenized N18TG2 cells and J774A.1 cells, which were treated for 24 h with heme (20 µM), as well as liver homogenates, prepared as described in the Material and Methods section. Heme oxygenase (HO) activity was determined in the presence of vehicle (DMSO), cannabidiol (CBD; 10 µM), or tetrahydrocannabinol (THC; 10 µM) as described in Material and Methods. Each data point represents the mean ± SD from two experiments (*n* = 2), which were measured in duplicates. Therefore, statistical analyses were not performed.

The reduced heme converting capacity in the presence of CBs might be the consequence of direct interaction with the enzyme in analogy to the (previously described) inhibition of cytochrome P450 isoenzymes in-vitro by CBD [67,68,69]. We, therefore, checked whether CBs are able to interfere with the HO reaction in-vitro directly. CBs were added straight to the assay performed with homogenates of neuroblastoma cells (N18TG2). The resulting HO activity was compared to the vehicle-treated control reaction. Both, CBD and to a much lesser degree THC, inhibited the in-vitro HO activity. This inhibition was not restricted to neuronal cells but was also found in homogenates from other cell types, such as liver tissue and macrophages (Figure 2).

**Figure 3:**
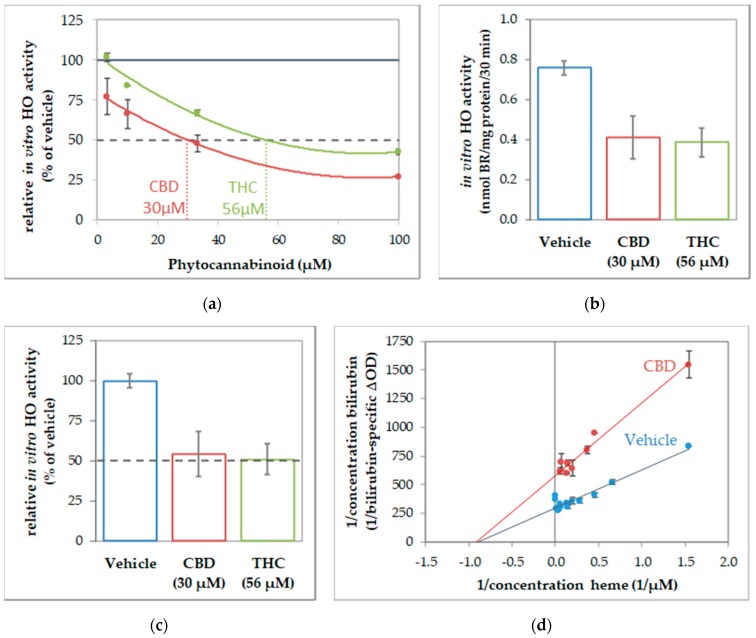
Cannabidiol acts as a non-competitive inhibitor of heme oxygenase. Determination of inhibitory concentration (IC) 50 of cannabinoids (CBs) inhibiting in-vitro heme oxygenase (HO) activity of J774A.1 cells. (**a**) Dilution series of cannabinoids (cannabidiol (CBD), red; tetrahydrocannabinol (THC), green) were used to inhibit the in-vitro HO activity of cell homogenate (J774A.1 cells) and calculated in % of the vehicle-treated control. The black dotted line indicate 50% inhibition. Values were obtained from duplicates and plotted against the input concentration of CBs. By polynomial analyses, the IC_50_ was calculated and the respective concentrations determined. (**b**,**c**) Verification of IC_50_. Determination of HO activity was performed in the presence of CBs at the calculated concentrations and results are shown (**b**) in nmol bilirubin (BR)/mg of protein/30 min and (**c**) in % of the vehicle control (set to 100%). The black dotted line indicates 50% inhibition. (**d**) The inhibitory characteristic of CBD was determined using a dilution series of heme in the presence of CBD (30 µM; red dots) or with vehicle (blue dots). The obtained values were plotted reciprocally. The regression analyses revealed a non-competitive inhibition mode of CBD on HO activity in cell homogenates. All experiments were performed once (*n* = 1) using duplicates/triplicates. Data are given as means ± SD.

To further characterize the interaction of CBs and HO, we next determined the enzyme kinetics in the absence and presence of CBs to characterize the inhibition mode. Given that CBs specifically inhibited HO activity in all investigated tissue material, we supposed that the inhibition mode of CBs should be independent of the cell type. Therefore, we used macrophages of the J774A.1 cell line for enzyme kinetic experiments, because this cell type showed the highest HO activity from all tissue material investigated, and is thus best suited for studying enzyme inhibition. First, we determined the IC_50_ of CBD and THC by using the dilution series of the respective CB (Figure 3). IC_50_ values of 30 µM for CBD and 56 µM for THC (Figure 3a) were calculated from the obtained dose-response curves by polynomial regression. We then verified that the calculated IC_50_ is indeed leading to an inhibition of 50% compared to the not-inhibited vehicle control, and found our calculation confirmed (Figure 3b,c). The type of inhibition was investigated for the more potent CB, CBD. We determined HO activity at increasing concentrations of the substrate hemin in the presence or absence of CBD at IC_50_ concentration. The product formation was displayed against the hemin amount in a double reciprocal fashion as the Lineweaver-Burk plot. The mode of inhibition exerted by CBD was found to be non-competitive (Figure 3d). 

All the experiments described above were repeated not more than two times, due to the limitation of the sample material. Therefore, we did not perform statistical tests to verify that the observed inhibitory effect of CBs on in-vitro HO activity is significant. Nonetheless, in the described different experimental approaches, we consistently found this inhibitory effect. Therefore, the cumulative evidence supports the conclusion that CBs possess the potential to act as an inhibitor of HO reaction. 

An inhibited HO reaction will result in lower levels of the heme degradation products BR and CO, which are critical for neuronal function and survival under stress conditions [20,21,22]. We, therefore, checked the neuronal survival and stress response elicited by the CBs in controls, and rotenone challenged primary mesencephalic cultures, and neuroblastoma cells. Further, we included BR as a supplemental treatment to overcome the potentially adverse effects of CBs due to an inhibited HO product formation.

### 3.2. Survival of Dopaminergic Neurons

**Figure 4:**
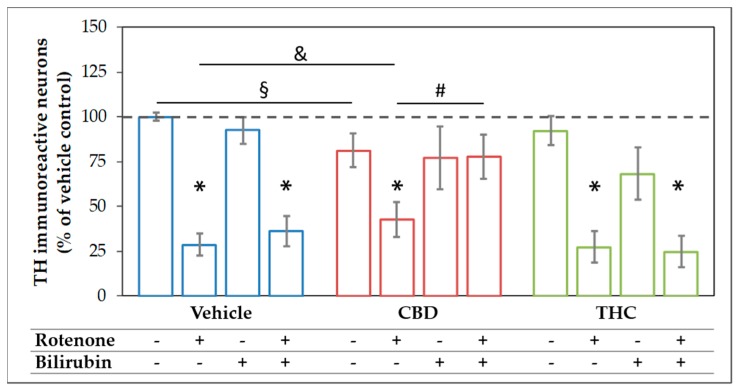
The combination of cannabidiol and bilirubin prevents rotenone-induced loss of neurons. Cells were treated with vehicle (DMSO), rotenone (80 nM), bilirubin (BR; 10 µM), cannabidiol (CBD; 10 µM), or tetrahydrocannabinol (THC; 10 µM) for 48 h. After tyrosine hydroxylase (TH) immunocytostaining, dopaminergic (TH immunoreactive, THir) cells were counted. Data are displayed as mean ± SEM calculated from eight independent experiments (*n* = 8). Statistical significance was determined using a Kruskal-Wallis test followed by a Mann-Whitney (U)-test. * indicates significant effects compared to the respective vehicle control (* *p* < 0.05). § indicates the effects vs. the respective group treated with CBs (§ *p* < 0.05). & indicates the effect of CBD supplementation on the rotenone treatment (& *p* < 0.05). # indicates the effects of BR supplementation (# *p* < 0.05). Exact *p* values for the group comparisons are shown in the supplementary material (Table S3).

**Figure 5:**
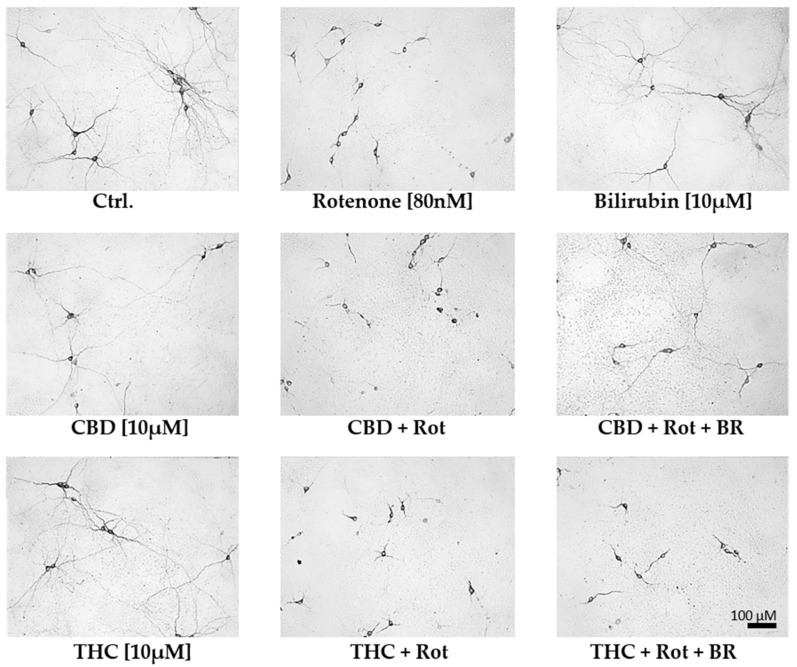
Cannabidiol and bilirubin in combination, partly prevent rotenone-induced morphological changes in neurons. Representative photographs were taken with 200× magnification and illustrate the morphology of dopaminergic neurons in primary mesencephalic cell culture on the 12th day in-vitro. Compared to vehicle (DMSO) control cultures (Ctrl.), rotenone (Rot; 80 nM) treated cells exhibit degenerative cell bodies, shorter neurites, and a lower number of primary neurites. Mesencephalic cells treated with 10 µM cannabidiol (CBD), tetrahydrocannabinol (THC), or bilirubin (BR) display a phenotype comparable to non-treated control (Ctrl.) neurons. Cells concomitantly treated with CBD, Rot, and BR have longer neurites than cells treated with CBD and Rot, but the number of primary neurites is still lower than in controls. Note that the number of dopaminergic neurons in the photographs does not fully reflect the overall number of cells in the entire culture dish.

The mesencephalic cultures used in this study comprise around 80–85% neurons, of which 1–2% were dopaminergic (Figure S3). Treatment with 10 µM CBD for 48 h significantly lowered the surviving dopaminergic neurons to 80% of the untreated control, while treatment with the same concentration of THC was without effects (Figure 4 and Figure 5). For BR, which is known to be neurotoxic at higher amounts [58], we found no effects on the number of dopaminergic neurons, N18TG2 cells, and astrocytes in concentration ranges from 0.001–10 µM (Figure 4 and Figure 5, Figure S4, Table S7). Rotenone (80 nM) was the only additive, which showed clear cytotoxic effects. The number of dopaminergic neurons was significantly decreased to 29% of the untreated vehicle control (Figure 4). Furthermore, the cell morphology was compromised as indicated by shorter neurites, lower numbers of primary neurites, and degenerated cell bodies (Figure 5) in primary mesencephalic cell preparations (Figure 4 and Figure 5). 

The rotenone-induced neurotoxic effect was partly reversed by CBD, which raised the amount of surviving dopaminergic neurons to 42% compared to the untreated control, but not by THC. BR-treatment could not counteract the CBD mediated loss of neurons, and in combination with THC, the number of surviving neurons tended to be even lower. Furthermore, BR neither alone nor in combination with THC was capable of protecting dopaminergic neurons against rotenone-mediated damage (Figure 4 and Figure 5). However, BR cooperated with CBD and strongly augmented the mild rescuing effect of CBD. Thus, the combination of both compounds, CBD and BR, was capable of fully preventing rotenone-induced loss of neurons. The numbers of surviving dopaminergic neurons were nearly identical to those seen in the CBD-only treated controls (Figure 4). Furthermore, CBD applied in combination with BR partly prevented the morphological changes induced by rotenone.

These data show that BR acts synergistically with CBD in protecting neurons against rotenone mediated cell stress and cell death in this culture system. These findings were supported by GFAP staining in mesencephalic cultures. The morphology of astrocytes was strongly affected by rotenone treatment and partially recovered by co-treatment with CBD and BR. So, the effect of BR alone or in combination with CBD in rotenone treated cells seems not to be neuron-specific (Figure S5, Table S7).

Nonetheless, primary mesencephalic culture systems comprise different cell types, which may all play a role in the underlying neuroprotective mechanism. In order to find out whether the synergistic protection of CBD and BR is operating directly in neurons, or involves other supportive cell types, we next focused our experiments on neuroblastoma N18TG2 cells, which consist exclusively of neurons. They were further applied to study CBD effects that are either receptor-mediated or direct by entering the cell and interacting with intracellular targets. Since we have shown that CBD treatment inhibited HO-activity in N18TG2 cells (Figure 2), we expected the protective mechanism to operate also in these cells. Additionally, using N18TG2 cells for these experiments also allowed us in adhering to the ethical commitment of our institution by reducing the number of animals.

### 3.3. Changes of Metabolic Activity of Neuroblastoma Cells upon Treatment 

We next verified that N18TG2 cells are suitable for elucidating the mechanism underlying CBD mediated protection against oxidative stress. Using the resorufin reduction assay, we found that the treatment with CBs alone did not affect the metabolic activity (Table 1). Similar to the data obtained for the dopaminergic neurons in mesencephalic cultures, we found that supplementation with BR significantly increased the metabolic activity in rotenone-stressed cells when this substance was applied in combination with CBD. The effect of BR on cells treated with rotenone or THC was not significant.

These data show that BR, in the presence of CBD, rescued N18TG2 cells stressed by rotenone, indicating that in this cell system, the HO reaction is also potentially beneficial against rotenone-induced stress and that BR cooperates with CBD in exerting neuroprotective effects. We, therefore, assumed that rescuing effects should also be reflected by the capability of the compounds to induce a cellular response to stress, and thereby, reward it. 

### 3.4. Determination of Treatment-induced Cell Stress Response Using Gene Expression

First, we tested the single compounds to elicit a cell stress response. Using qPCR, we analyzed different sets of markers typically associated with pro-oxidative and injury-associated stress [70,71]. 

**Figure 6:**
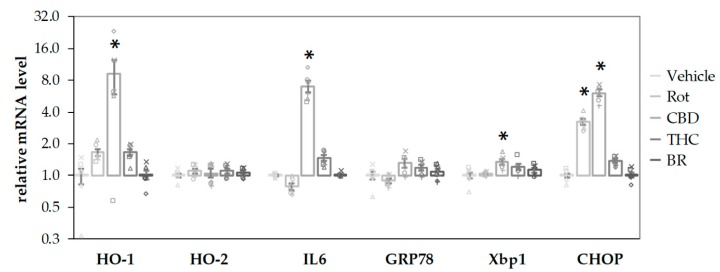
CBD and rotenone induce cellular stress responses in N18TG2 cells. N18TG2 cells were treated with vehicle (DMSO), rotenone (Rot; 80 nM), bilirubin (BR; 10 µM), cannabidiol (CBD; 10 µM), or tetrahydrocannabinol (THC; 10 µM) for 16 h. Expression levels of mRNA for heme oxygenase 1 and 2 (HO-1; HO-2), interleukin 6 (IL6), glucose-regulated protein 78 (GRP78), X-box protein 1 (Xbp1), and C/EBP homologous protein (CHOP) were determined by quantitative reverse transcription PCR. Values were normalized to the internal reference gene Cyclophilin A, the obtained data were expressed relative to the respective control and are displayed on a logarithmic scale with the base of 2. Data are shown as both, individual values and mean ± SEM of six experiments (*n* = 6) performed in duplicates. One-way ANOVA, followed by Bonferroni was performed to calculate significant differences compared to the vehicle control and are indicated (* *p* < 0.05). Exact *p* values for the group comparisons are shown in the supplementary Table (Table S5).

CBD stimulated the mRNA expression of the stress-associated genes, HO-1, IL6, and XBP1 (an early endoplasmatic reticulum (ER) stress marker) after a 16-h incubation period (Figure 6). Both CBD and rotenone increased C/EBP homologous protein (CHOP) mRNA, a marker for activation of cell death pathways, indicating harmful effects elicited by the compounds. In contrast, neither BR nor THC affected gene expression at all. None of the additives induced significant changes for the mRNA of HO-2, the constitutive HO isoform, and GRP78, an ER stress marker downstream of XBP1 splicing. The stress response elicited by CBD could be interpreted as a consequence of the inhibited HO reaction and the subsequently lower capability to cope with cell stress, in particular with oxidative stress. 

**Figure 7:**
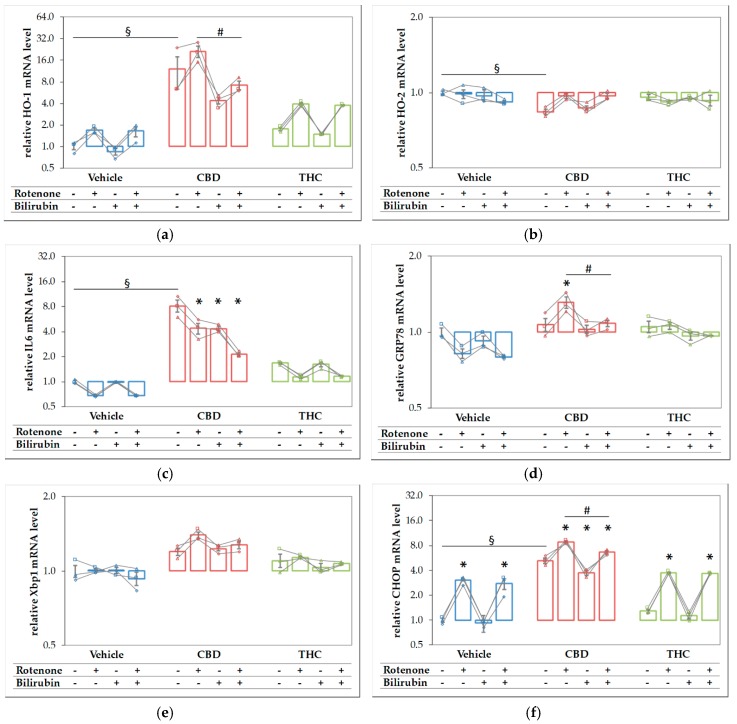
Rotenone and cannabidiol act synergistically in eliciting a stress response. N18TG2 cells were seeded 2 h before experiment and treated with vehicle, cannabidiol (CBD; 10 µM) or tetrahydrocannabinol (THC; 10 µM) alone and in combination with rotenone (Rot; 80 nm) or BR (10 µM) for 16 h. Gene expression levels of (**a**) the inducible isoform of heme oxygenase (HO-1), (**b**) the constitutive isoform of HO (HO-2), (**c**) interleukin 6 (IL6), (**d**) glucose-regulated protein 78 (GRP78), (**e**) X-box protein 1 (Xbp1), and (**f**) the pro-apoptotic marker C/EBP homologous protein (CHOP) were determined by reverse transcription-quantitative PCR, normalized to the internal reference gene Cyclophilin. Data are shown as both individual values and mean ± SEM of three biological replicates (*n* = 3) and are displayed relative to the untreated control condition (vehicle control), which was set to 1. The obtained data are displayed on a logarithmic scale with the base of 2. One-way ANOVA, followed by Bonferroni was performed to calculate the differences between groups. Values of *p* ≤ 0.05 were considered significant, and the following symbols indicate significant differences to the vehicle control group (§), to the respective control group (*), and between the condition with added Rot vs. Rot + BR (#). Exact *p* values for the group comparisons are shown in the supplementary material (Table S6).

Since both compounds, CBD and rotenone, were able to elicit a stress response by themselves, we expected that the combined treatment should result in an even higher stress level, and thus, in a further increase of the respective target mRNAs. We, therefore, analyzed the expression of the stress genes shown in Figure 6 in N18TG2 cells treated with the substances in combination and compared these effects to those elicited by the single compounds (Figure 7). Similar to the results shown in Figure 6, CBD alone resulted in higher mRNA expression levels of HO-1 (Figure 7a), IL-6 (Figure 7c), and CHOP (Figure 7f), and additionally in a slightly lower expression of HO-2, while THC showed no effects. Except for IL6, which was decreased, we found significantly higher expression levels of all targets, when CBD was added together with rotenone. This indicates that the stress elicited by rotenone is enhanced when CBD is co-applied, suggesting additive effects of both stressors. Adding BR to the CBD-treated cells resulted in significantly decreased mRNA expression of HO-1, GRP78, and CHOP (Figure 7a,d,f). BR, in combination with CBD, nearly completely reverted the rotenone-mediated stress response for the affected targets, and resulted in even lower levels of HO-1 and IL6 than those observed for CBD alone (Figure 7a,c). In contrast to CBD, BR had no effect on rotenone-induced effects when applied in combination with THC.

In order to further analyze the association of the CB-mediated protection against rotenone-induced toxicity and the role of BR in this setting, we performed univariate analyses of variance and determined the respective interactions using alpha-values. To obtain insight, whether an interaction between CBD and BR could explain the observed synergistic effects, we limited these analyses to rotenone treated groups only (Table 2). 

The survival of dopaminergic neurons was compromised by rotenone. In contrast to THC, CBD and BR had the potential to increase the survival of rotenone-stressed neurons (see Figure 4 and Figure 5, Table 1 and Table 2). The data displayed in Table 2 show that all compounds had the capability to modulate the expression of stress response genes. However, BR operated in directions opposite to the other additives. Both CBs increased the expression of stress genes when co-administered with rotenone, while BR counteracted the rotenone mediated effects. Further, BR showed a significant interaction with CBD on many stress markers, which was absent for THC.

**Figure 8:**
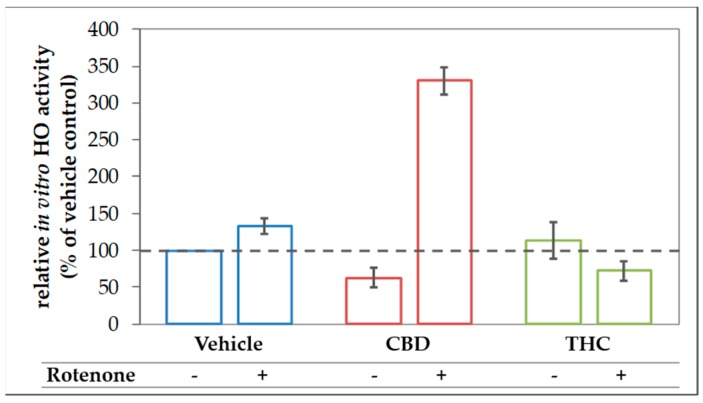
CBD boosts in vitro HO activity in rotenone treated N18TG2 cells. N18TG2 cells were seeded in 12-well plates and incubated with vehicle (DMSO), rotenone (80 nM), with cannabidiol (CBD; 10 µM), or tetrahydrocannabinol (THC; 10 µM) for 48 h. HO activity was determined as described in Material and Methods and calculated relative to the vehicle control, which was set to 100% (dashed black line). Samples were analyzed in duplicates, and the experiment was repeated twice (*n* = 2). Data are shown as mean ± SD. Due to the low number of biological replicates, a statistical examination was not performed.

The finding of the increased expression of stress response genes, and in particular, the increased HO-1 expression in rotenone-stressed cells treated with CBD can be interpreted as an attempt to strengthen the cellular defense against (oxidative) stress. Consequently, the induction of HO-1 is expected to increase the cellular capacity to convert heme. However, to overcome the inhibition of the HO enzyme reaction exerted by CBD, which we have described in Section 3.1, the actual heme degradation capacity should be even higher, when an additional stressor, such as rotenone, is applied. Therefore, we next examined the HO activity in N18TG2 cells incubated with CBD or THC in combination with rotenone. As expected, CBD treatment augmented the heme converting capacity by more than three-fold in rotenone-stressed cells (Figure 8). 

## 4. Discussion

Despite the vast literature on the effects exerted by CBs [54,56,72], the mechanisms underlying neuroprotective effects are still elusive. In this study, we aimed at elucidating the mechanism underlying the capability of CBs to elicit a stress response and simultaneously confer protection against oxidative stress. To that aim, we used an in-vitro system, consisting of primary mesencephalic neurons, in which we were able to previously show that CBD exerts protective effects against MPP^+^ generated stress [7]. In this study, we used rotenone to generate mitochondria-derived oxidative stress. Rotenone is a proven inhibitor of complex I of the mitochondrial electron transport chain leading to a blockade of oxidative phosphorylation with reduced synthesis of ATP [73]. Additionally, blocked electron transfer to oxygen leads to ROS-formation, especially superoxide radicals, oxidative DNA damage, and the subsequent induction of apoptosis [74]. 

We found that treatment with rotenone not only resulted in lower numbers of surviving neurons with degenerated morphology in our preparations of mesencephalic cells (Figure 4 and Figure 5) but was also leading to an increased mRNA expression of CHOP, also known as growth arrest- and DNA damage-inducible gene 153, in N18TG2 neuroblastoma cells (Figure 6 and Figure 7). Upregulation of the pro-apoptotic transcription factor CHOP, therefore, indicates stressful conditions associated with imbalances of the redox homeostasis that are sensed by the endoplasmic reticulum [75]. These findings show that our model is suitable to investigate the mechanism underlying the presumed neuroprotective effects afforded by CBs under rotenone-induced stress. We hypothesized that the protective mechanism involves the HO system that plays a key role in decreasing oxidative stress [19,20,21,22,23]. We, therefore, tested whether CBs are capable of upregulating HO activity, thereby leading to enhanced production of BR, a well-recognized neuroprotectant [21,39,55]. 

### 4.1. Reduction of In-Vitro Heme Converting Capacity by CBs

Our data strongly suggest that CBs are capable of inhibiting HO activity. Most experiments, in which we determined HO activity, were only repeated twice. We, therefore, restrained from performing statistical analyses. However, the HO inhibiting effect of CBs was reproducible in different experimental approaches and systems. All these data suggest that the inhibition of the enzymatic HO reaction represents an intrinsic property of THC and CBD, with CBD being more potent.

Treatment of neuronal cells for 48 h with 10 µM CBD appeared to nearly halve HO activity displayed by the cell homogenates (Figure 1 and Figure 8). We cannot absolutely exclude that the observed inhibition was due to CBs remaining adhered to the cell membrane after the incubation period and contaminating the cell homogenates. We therefore tested whether adding CBs straight to the biochemical HO assay would affect the in-vitro enzyme reaction. We found that CBD and THC appear to inhibit the enzyme reaction directly and that this inhibition is not restricted to neuronal cells, but possibly also operating in each cell type (Figure 2). We could further demonstrate that CBD inhibits the HO reaction in a non-competitive fashion (Figure 3). The obtained inhibition constants were relatively similar in all the tested homogenates. Considering that CBs are able to enter the cell, they may access the HO enzyme and directly exert inhibitory effects. Such direct interaction with the HO enzyme has not been described so far. However, both CBD and THC have been shown to inhibit the activity of several other enzymes. Bartova and Birmingham [76] have reported that THC inhibited mitochondrial NADH-oxidase activity in homogenates from different brain regions of the rat. THC at 10 µM inhibited NADH-oxidase activity, between 70–50%, demonstrating that the effects are in a range comparable to those found in our study. In addition, cytochrome P450 enzymes are influenced by CBs with CBD being the most potent inhibitor [14]. HO and P450 are both heme enzymes [77], which may suggest a similar mechanism of inhibition exerted by CBs. 

### 4.2. Induction of Cell Stress Response by CBs

The fact that CBD (and to a lesser extent THC) is able to inhibit the conversion of heme may explain the findings of an increased stress response, which has also been reported by others [47,48]. CBD, in contrast to THC, elicited a clear stress response characterized by the upregulation of IL6 and the pro-apoptotic marker CHOP, supporting the findings that CBD acts as a stressor itself. Our findings are also in accordance with a comparative gene expression profiling study by Juknat et al. [47] using the BV-2 microglial cell line showing that CBD induced a robust transcriptional response of genes associated with enhanced oxidative stress and GSH deprivation involving the Nrf2 and ATF4 transcription factors. Another study showed that CBD mediated an increase in HO-1 protein in smooth vascular cells that could be reversed by the GSH precursor N-acetylcysteine, indicating the participation of ROS signaling and enhanced oxidative stress in this system as well [48]. 

Various models have shown that experimental inhibition of HO activity by different means augments oxidative stress and increases the expression of stress response genes [24,25,26,27]. Thus, it is conceivable that CBD mediated moderate death rate of dopaminergic neurons, and the CBD-induced stress response, which we have determined in our model systems, are at least partially based on the inhibition of HO and the decreased formation of HO products, such as BR.

We have shown that CBs are capable of modulating the HO system not only in neurons but also in different cell types. The application of compounds that inhibit HO activity is therapeutically interesting as anti-cancer drugs. Many tumor types benefit from upregulated HO-1, and a subsequently increased formation of CO or BR. Therefore, newly designed drugs capable of inhibiting HO activity in-vivo yielded promising results [78,79,80,81]. Interestingly, CBs have been shown to exert distinct antitumor effects (for review, see [82]). In view of our data, it is tempting to speculate that modulation of the HO system may represent a new potential mechanism supporting the therapeutic effects of CBs.

It should be underlined that the mode of inhibition exerted by a compound has to be taken into account for understanding such an in-vivo effect. A competitive inhibition causes a substantial drop in enzyme activity at low substrate concentrations [83]. Thus, substrate accumulating over time will overcome the inhibition by two synergistically operating effects; the decreased inhibition seen at elevated substrate concentrations and the super-induction of the enzyme due to accumulating substrate, as was described [84]. In previous experiments, we have shown that long term incubation with the reversible competitive HO inhibitor Zn (II) protoporphyrin IX leads to a compensatory upregulation of HO-1, and subsequently, an increased heme degradation capacity, when the amount of enzyme molecules increases sufficiently [59].

In contrast, for a non-competitive inhibitor, opposite effects will occur. While at low substrate concentrations, the inhibitory effect is minor, inhibition increases with increasing substrate concentrations until being maximal [83]. Considering that in the absence of additional stress, the substrate heme does not accumulate and the demand for heme degradation products is low, increased production of enzyme molecules is not required under these conditions. Further, since the inhibition exerted by a non-competitive inhibitor, such as CBD, is minimal at low substrate concentrations, the existing amount of HO enzyme molecules may suffice to overcome this inhibition. Nevertheless, upon prolonged incubation with CBD stress may occur in sensitive cells, which suffer from low antioxidative capacity, such as neurons, and result in a subsequent stress response. 

### 4.3. Acceleration of in Vitro Heme Converting Capacity by Treatment with CBs and Rotenone

Since rotenone inhibits mitochondria in terms of ATP production and leads to an increased generation of ROS, its application increases the demand for HO products. Therefore, the effect of CBD-mediated HO inhibition will result in further increased stress, triggering HO-1 induction. HO inhibition or silencing has been shown to sensitize cells towards an additional (oxidative) stressor [85,86]; but at the same time inhibition of HO activity acts as a HO-1 inducing stimulus [84]. Such stress-triggered super-induction may increase stress tolerance in surviving cells via HO super-induction. We found that HO-1 mRNA levels were higher when cells were treated simultaneously with rotenone and CBD (Figure 6), suggesting increased cell stress. We further found that co-treatment of N18TG2 cells with CBD and rotenone resulted in an enormous increase in the cellular heme degradation capacity under substrate saturating conditions of our in-vitro HO assay. These results indicate that CBD-mediated HO inhibition could be overcome, and suggest that the combined treatment of CBD and rotenone resulted in the super-induction of HO (Figure 8). However, despite the increased heme degrading capacity, we found that BR is required to assist CBD for exerting full protection against rotenone-induced cell stress. Supplementation of the CBD-treated rotenone stressed mesencephalic cells with BR also increased the number and morphology of surviving neurons to nearly the values of the control group (Figure 4 and Figure 5). This suggests an important role for HO/HO products in CBD mediated neuroprotection. The strongly enhanced heme degradation capacity, at the first view, argues against the additional requirement of the heme degradation product BR. It is possible that the required high levels of BR cannot be met under the cell culture conditions we have applied. However, when higher amounts of the HO substrate heme are available, the resulting amounts of BR may be sufficient for providing cell protection (see Supplemental Figure S6). BR is transported by albumin and plasma levels of BR are higher than those used in our study. In healthy humans, levels of unconjugated BR in plasma reach 20 µM [87]. Unconjugated BR can pass the blood-brain barrier to a certain extent, as has been shown in a mouse model of neurotoxicity induced by excessive BR applied via the tail vein [88]. Therefore, it is possible that BR levels *in-vivo* are sufficient to supplement the neuroprotective effects of CBD. 

Interestingly, BR was not able to rescue rotenone-treated neurons in the absence of CBD. Both, BR and CBD are effective by interacting against rotenone-induced cell stress, as shown in Table 2. These data suggest that neuroprotection exerted by BR, which has been described in many studies [21,39,55], is a supplementary effect. It may require additional factor(s), such as CBD, rendering BR fully efficient. Further studies will be required to uncover the pathways elicited by CBD, which renders the BR-mediated protection fully efficient.

**Figure 9:**
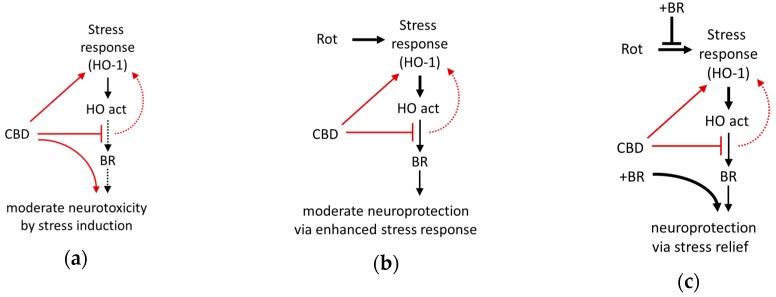
Schematic presentation of the suggested mechanism underlying neuroprotection mediated by the interaction of bilirubin with cannabidiol against cell stress investigated in mesencephalic cultures. (**a**) The phytocannabinoid cannabidiol (CBD) itself exerts cell stress (and moderate death of neurons), probably due to the inhibition of the heme oxygenase (HO). (**b**) In combination with enhanced oxidative cell stress caused by rotenone (Rot), CBD acts as a booster for the stress response and super-induces HO-1, resulting in an increased cellular capacity to convert heme (HO activity), which is associated with a slight increase of surviving neurons. However, the levels of bilirubin (BR) produced appear to be insufficient (CBD inhibits HO activity), and only (**c**) exogenous supplementation with BR fully restores neuronal survival. Additionally, BR supplementation abolishes Rot-induced expression of the stress marker (HO-1 mRNA), which can be taken as a sign of reduced cell stress. Red lines and arrows indicate direct effects exerted by CBD; black lines indicate effects or pathways. Dotted lines indicate presumed (red)/insufficient effects (black).

Our data indicate that BR and CBD exert protection against rotenone-mediated neuronal death by a cooperative mechanism (Figure 9). CBD exerts a cell stress response, which may be caused by the inhibition of the HO enzyme. BR counteracts the cell stress response and appears to exert neuroprotection by supplementing BR levels produced in the presumably ineffective HO reaction. Collectively the data presented in this study suggest that the HO system is relevant for CBD mediated cytoprotection. They further indicate that neuronal protection against adverse conditions at least partly involves changes in the HO system to which the initial HO-inhibition exerted by CBD may contribute. The degree of protection, however, appears to critically depend on endogenous BR levels. Further studies are required to elucidate the precise mechanism underlying the exclusive cooperative effect of BR and CBD on neuroprotection.

## 5. Conclusions

We could show that CBD (10 µM) can counteract rotenone (80 nM) induced neuronal damage in primary cultures, possibly because this CB itself induces cell stress, which is resulting in mild neurotoxicity in our in-vitro system. The neuroprotection is profoundly enhanced by co-treatment with BR (10 µM), pointing towards the critical involvement of HO. The newly discovered non-competitive inhibition of the HO reaction exerted directly by CBD may represent a hitherto unknown mechanism involved in stress adaptation. CBD, possibly by inhibiting the HO reaction, acts as an enhancer of the cell stress response (Figure 9). Similar to other conditioning mechanisms, CBD may provide delayed cytoprotection due to its capability to upregulate the stress response. Our findings further suggest that CBD via HO may confer full protection against (oxidative) stress in-vivo where endogenous levels of BR are sufficiently high.

## Figures and Tables

**Table 1:** Bilirubin increases the metabolic activity of rotenone-stressed N18TG2 cells co-treated with cannabidiol.

	Veh	CBs	CBs + Rot	CBs + Rot + BR
CBD	100.00	103.88	61.42 *	76.07 * #
SEM	0.41	3.05	2.30	1.61
THC	100.00	110.46	88.35 *	90.95 *
SEM	1.16	4.22	3.77	1.59

Cells were treated for 48 h with the compounds as described in the Material and Methods. Data present the mean and the SEM of seven independent resazurin reduction experiments performed in duplicates in the presence of the cannabinoids (CBs), tetrahydrocannabinol (THC; 10 µM), or cannabidiol (CBD; 10 µM) and rotenone (Rot; 80 nM) and bilirubin (BR; 10 µM). For a paired comparison between CBs vs. CBs + Rot treatment (* *p* < 0.05), and CBs + Rot vs. CBs + Rot + BR treatment (# *p* < 0.05), the Mann-Whitney (U)-test was used. Bold letters indicate significant differences. Exact *p* values for the group comparisons are shown in the supplementary material (Table S4).

**Table 2:** Main effects of single compounds and interaction of cannabidiol (CBD), tetrahydrocannabinol (THC), or bilirubin (BR) with rotenone-induced toxicity and the cooperation of BR with CBD on rotenone-mediated effects after incubation for 24 h (for gene expression) and 48 h (for dopaminergic neuron survival) on the respective dependent variables.

Variable	Main Effect (*p* Value)				Main effect on Rotenone (*p* Value)			Interaction^1^ of BR on CBD (α Value)	Interaction ^1^ of BR on THC (α Value)
	Rotenone	CBD	THC	BR	CBD	THC	BR		
**Survival**	**↓<0.001**	0.374	0.051	0.196	↑**<0.001**	0.392	↑**0.024**	0.137	0.575
mRNA									
HO-1	0.072	↑**<0.001**	↑**<0.001**	↓**0.006**	↑**<0.001**	↑**<0.001**	↓**0.006**	↓**0.007**	0.951
HO-2	0.060	↓**0.01**	0.188	0.442	0.562	0.922	0.281	0.264	0.271
IL6	↓**<0.001**	↑**<0.001**	↑**<0.001**	↓**0.001**	↓**<0.001**	↓**0.001**	↓**0.01**	↓**0.01**	0.636
CHOP	↑**<0.001**	↑**<0.001**	↑**0.001**	↓**<0.001**	↑**<0.001**	↑**0.037**	↓**0.003**	↓**0.01**	0.926
GRP78	↑**<0.001**	↑**<0.001**	↑**<0.001**	↓**<0.001**	↑**<0.001**	↑**0.005**	↓**0.013**	↓**0.035**	0.857
XBP1	0.156	↑**<0.001**	↑**0.004**	0.2	↑**<0.001**	0.255	↓**0.046**	0.559	0.987

^1^ Interaction was analyzed in rotenone-treated groups only. While the survival of dopaminergic neurons (DN survival) was determined in mesencephalic cultures, all other variables were determined in N18TG2 cells. The interaction is considered significant if alpha is less than 0.05. ↑ indicates that the corresponding variable is increased compared to the untreated group. ↓ indicates that the corresponding variable is decreased compared to the untreated group. *p* values are highlighted in bold, when significant changes were observed (One-way ANOVA followed by Bonferroni per treatment regime).

## Supplementary Materials

The following are available online at https://www.mdpi.com/2076-3921/9/2/135/s1, **Figure S1**: No loss of BR during incubation of samples subjected to the in-vitro HO assay with tissue homogenate. Tissue samples (liver tissue, blue; brain tissue, grey) were prepared as described for liver tissue in Material and Methods section. Samples were assembled as described for the HO assay, except th at BR (30 pmol) was added instead of hemin. BR was extracted after incubating the samples on ice (0 °C, light blue, light grey) or at 37 °C (intense blue, intense grey). BR was quantified by means of spectrophotometry, as described in the Material and Methods section, and the obtained results were expressed relative to the 0 °C control determined without homogenate. The experiment was performed once; **Table S1**: Information about Intron-spanning primers; **Table S2**: Optimised protocol and validation studies using cDNA pool (Cyclo, HO-1, CHOP, Xbp1, GRP78) or amplificate (IL6, iNOS) dilution series; **Figure S2**: Performance of qPCR assays for Cyclo (**a**–**c**), HO-1 (**d**–**f**), CHOP (**g**–**i**), Xbp1 (**j**–**l**), GRP78 (**m**–**o**), IL6 (**p**–**r**) and HO-2 (**s**–**u**) was verified in separate experiments by performing a dilution series of a cDNA pool (**a**–**o**,**s**–**u**) or PCR product (**p**–**r**). In (**b**,**e**,**h**,**k**,**n**,**q**,**t**) amplification plots and (**c**,**f**,**i**,**l**,**o**,**r**,**u**) melt curve samples are shown in green while controls (no reverse transcription control (NRT) and no template control (NTC)) are shown in yellow and orange respectively; **Table S3**: Exact *p* values for the data shown in Figure 4; **Table S4**: Exact *p* values for the data shown in Table 1; **Table S5**: Exact *p* values for the data shown in Figure 6; **Table S6**: Exact *p* values for the data shown in Figure 7; **Figure S3**: Immunocytostaining against the glial fibrillary acidic protein (GFAP; 1:1000, Millipore, Germany), tyrosine hydroxylase (TH), and the neuronal nuclei marker NeuN (1:1000, Millipore, Germany) in mesencephalic cultures. Anti-GFAP and -NeuN staining (both 1:1000, Merck Millipore, Germany) was done according to the method described in Section 2.1.3. The number of dopaminergic neurons represents less than 1% of the total cell population; **Figure S4**: Effects of bilirubin on N18TG2 and U87 cells or dopaminergic neurons in primary cultures. Cells were treated with different concentrations of bilirubin (BR) for 48 h. Neuroblastoma and astrocytoma cells were stained with crystal violet (**a**,**b**), and dopaminergic neurons in mesencephalic primary cultures for tyrosine hydroxylase (TH, **c**) before counting. Data are shown as mean ± SEM of four (**a**,**b**) of five (**c**) independent experiments, respectively. No statistical significance was detected using the Kruskal–Wallis (H) test; **Table S7**: Effect of bilirubin on resazurin formation; **Figure S5**: Glial fibrillary acidic protein (GFAP) immunocytochemistry in mesencephalic cultures. Anti-GFAP staining (both 1:1000, Merck Millipore, Germany) was done according to the method described in Section 2.1.3; **Figure S6**: CBD accelerates BR formation from heme by an increased HO reaction. N18TG2 cells were treated with heme (10 µM) with or without adding CBD (10 µM). The formed BR was extracted from the cell culture medium after 50 h. One-hundred and fifty µL of medium was supplemented with 50 µL caffeine solution and 50 µL KCl, and the samples treated as the samples obtained in the HO-assay, as described in the Material and Methods section. The amount of BR in benzene-extracts, determined by spectrophotometry, was corrected for the underlying cell number (blue bars, primary Y-axis). Cell number was determined using the crystal violet assay and calculated as cell equivalent (violet open diamond, secondary Y-axis) given relative to the control cells, which were treated by vehicle only and set to 1 (veh control). The BR extracted from the control cells was below the detection limit. Data from one experiment performed in triplicates are given as means ± SD.
