# Supplementary material for: Cannabidiol Protects Dopaminergic Neurons in Mesencephalic Cultures against the Complex I Inhibitor Rotenone Via Modulation of Heme Oxygenase Activity and Bilirubin
Source: Antioxidants (Basel). 2020 Feb 4;9(2):135. doi: 10.3390/antiox9020135 (PMC7070382; doi:10.3390/antiox9020135)
Supplement: Supplementary file 1 [file antioxidants-09-00135-s001.pdf]

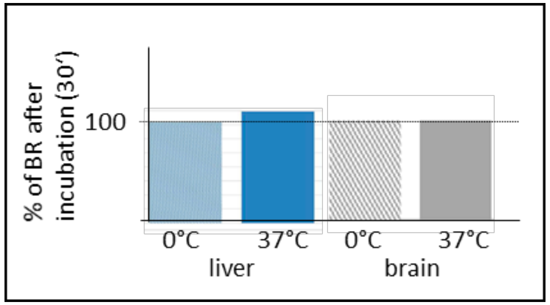

Figure S1. No loss of BR during incubation of samples subjected to the *in-vitro* HO assay with tissue homogenate. Tissue samples (liver tissue, blue; brain tissue, grey) were prepared as described for liver tissue in Material and Methods section. Samples were assembled as described for the HO assay, except that BR (30 pmol) was added instead of hemin. BR was extracted after incubating the samples on ice (0 °C, light blue, light grey) or at 37 °C (intense blue, intense grey). BR was quantified by means of spectrophotometry, as described in the Material and Methods section, and the obtained results were expressed relative to the 0 °C control determined without homogenate. Experiment was performed once.

Table S1. Information about Intron-spanning primers.

Target	Accession number	Sequence	Position on plus strand	Product-length (bp)	Exon junctions in	Intron size (bp)	Source
**Cyclo**	NM_008907.1	CAT GGC AAA TGC TGG ACC AAA<br>TGC CTT CTT TCA CCT TCC CAA A	338<br>447	110	product	192	newly designed
**HO1**	NM_010442.2	**CCT TCC CGA ACA TCG ACA GCC**<br>**GCA GCT CCT CAA ACA GCT CAA**	635<br>784	150	reverse primer	---	Zhao et al. (2006)
**CHOP**	NM_007837.4	**CCC TGC CTT TCA CCT TGG**<br>**CCG CTC GTT CTC CTG CTC**	181<br>551	371	product	244	Hosoi et al. (2012)
**Xbp1 (s/us)**	NM_013842.3	**GAA CCA GGA GTT AAG AAC ACG**<br>**AGG CAA CAG TGT CAG AGT CC**	720<br>924	205	product	771	Pfaffenbach et al. (2012)
	NM_001271739.1	**GAA CCA GGA GTT AAG AAC ACG**<br>**AGG CAA CAG TGT CAG AGT CC**	720<br>898	179	product	797	
**GRP78**	NM_001163434.1	**TCT CCA CGG CTT CCG ATA AT**<br>**GTA CCT TTG TCT TCA GCT GTC ACT C**	1505<br>1706	202	product	317	Wey et al. (2012)
**IL6**	NM_031168.2	**GAG GAT ACC ACT CCC AAC AGA CC**<br>**AAG TGC ATC ATC GTT GTT CAT ACA**	187<br>327	141	product	1271	Guiletti et al. (2001)
**HO2**	NM_001136066.2	**GGA CAA TGC CCA GCA ATT CA**<br>**CTG CCT CCT AGT GTA CCT TTG TC**	720<br>983	264	product<br>reverse primer	665<br>---	newly designed

Table S2. Optimised protocol and validation studies using cDNA pool (Cyclo, HO1, CHOP, Xbp1, GRP78, HO2) or amplificate (IL6) dilution series.

Target	Annealing Temp (°C)/Time (sec)	Extension Temp (°C)/Time (sec)	ΔCt (RT + to RT-)	slope	Correlation Coefficient (Pearson) R^2	Verified Dynamic Range
**Cyclo**	62/20	72/20	8,80	–3,463	0,997	10^5
**HO1**	62/20	72/20	13,76	–3,414	0,996	4^5
**CHOP**	62/20	72/20	16,13	–3,245	0,991	2^6
**Xbp1**	62/20	72/20	10,76	–3,120	0,994	4^5
**GRP78**	62/20	72/20	10,24	–3,216	0,998	10^5
**IL6**	62/20	72/20	9,04	–3,229	0,999	10^7
**HO2**	62/20	72/20	16,85	–3,432	1,000	4^6

ΔCq gives the lowest difference in between Cq of sample and Cq of corresponding no reverse transcriptase control measured throughout this study.
T_{melt}: melting temperature

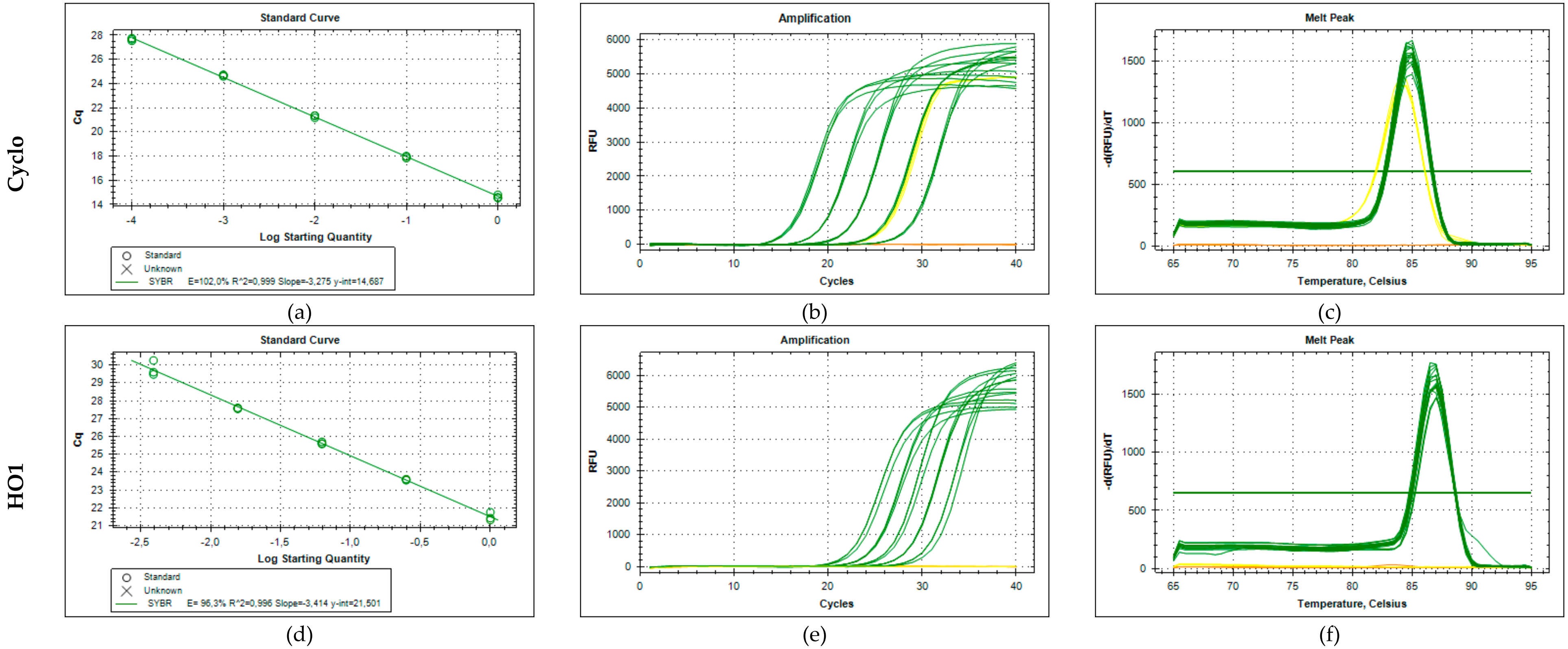

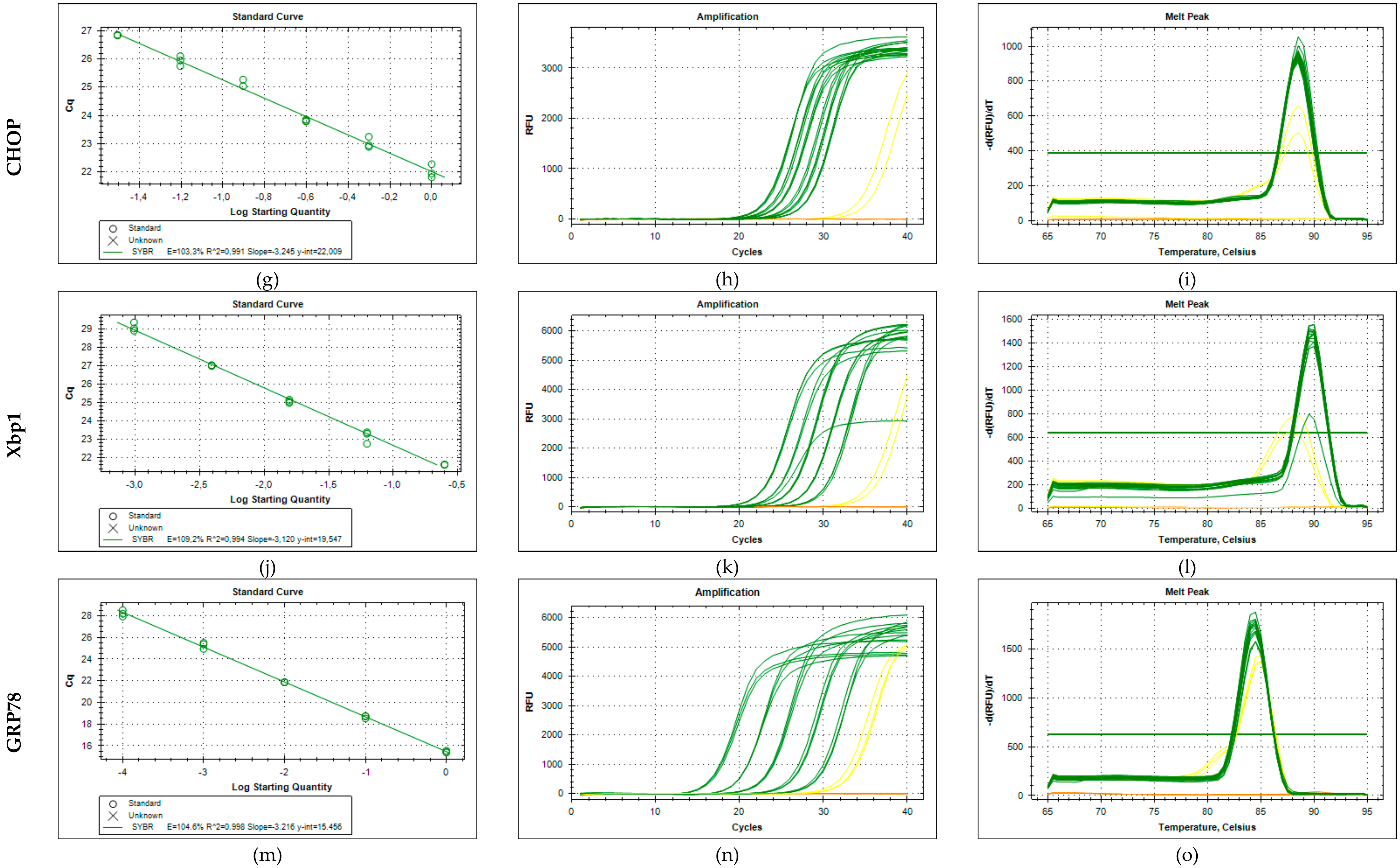
CHOP
Xbp1
GRP78
Standard Curve
Cq
Log Starting Quantity
Standard
Unknown
SYBR E=103,3% R^2=0,991 Slope=-3,245 y-int=22,009
SYBR E=109,2% R^2=0,994 Slope=-3,120 y-int=19,547
SYBR E=104.6% R^2=0.998 Slope=-3.216 y-int=15.456
Amplification
RFU
Cycles
Melt Peak
-d(RFU)/dT
Temperature, Celsius
(g)
(h)
(i)
(j)
(k)
(l)
(m)
(n)
(o)

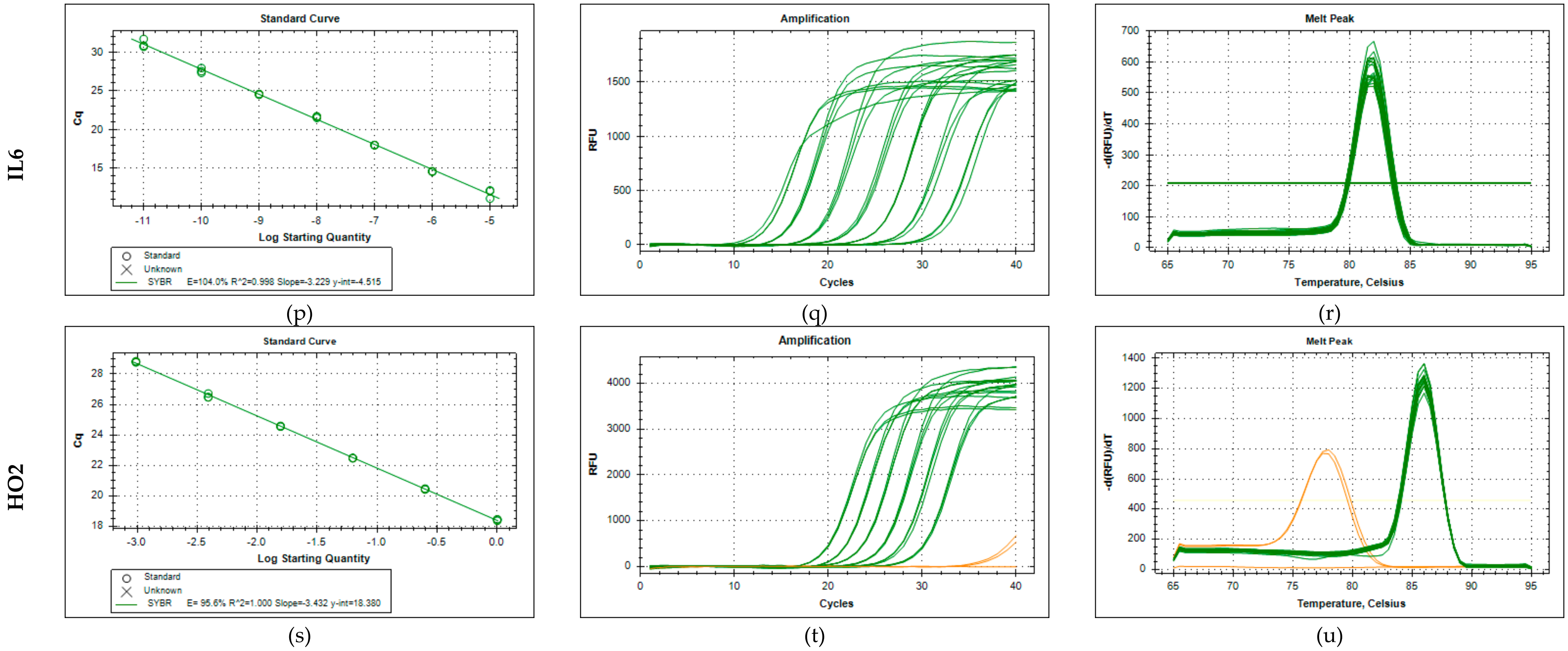

Figure S2. Performance of qPCR assays for Cyclo (**a, b, c**), HO1 (**d, e, f**), CHOP (**g, h, i**), Xbp1 (**j, k, l**), GRP78 (**m, n, o**), IL6 (**p, q, r**) and HO2 (**s, t, u**) was verified in separate experiments by performing a dilution series of a cDNA pool (**a-c, d-f, g-i, j-l, m-o, s-u)** or PCR product **(p-r**). In (**b, e, h, k, n, q, t**) amplification plots and (**c, f, i, l, o, r, u**) melt curve samples are shown in green while controls (no reverse transcription control (NRT) and no template control (NTC)) are shown in yellow and orange respectively.

Table S3. Exact p values for the data shown in figure 4.

			Neuronal Survival
§	Vehicle vs.	CBD	**0,001**
		THC	0.241
*	Vehicle vs.	Vehicle + Rot	**<0.001**
		Vehicle + BR	0.717
		Vehicle + BR +Rot	**<0.001**
#	Vehicle + Rot vs.	Vehicle + BR +Rot	0.307
*	CBD vs.	CBD + Rot	**0,002**
		CBD + BR	1.000
		CBD +BR + Rot	0.402
#	CBD + Rot vs.	CBD + BR + Rot	**0,008**
*	THC vs.	THC + Rot	**<0.001**
		THC + BR	0.07
		THC + BR + Rot	**<0.001**
#	THC + Rot vs.	THC + BR + Rot	0.807

Kruskal-Wallis test followed by Mann-Whitney-U (SPSS); signs used to display the significances are given in the first row. p<0.05 were considered significant and are highlighted in bold.

Table S4. Exact p values for the data shown in table 1.

			Metabolic Activity
*	CBD vs.	CBD + Rot	**<0.001**
		CBD + BR + Rot	**<0.001**
#	CBD + Rot vs.	CBD + BR + Rot	**0.004**
*	THC vs.	THC + Rot	**0.004**
		THC + BR + Rot	**0.019**
#	THC + Rot vs.	THC + BR + Rot	0.587

Kruskal-Wallis followed by Mann-Whitney-U (SPSS); signs used to display the significances are given in the first row. p<0.05 were considered significant and are highlighted in bold.

Table S5. Exact p values for the data shown in Figure 6.

		HO-1	HO-2	IL6	GRP78	Xbp1	CHOP
Vehicle vs.	Rotenone	1.000	1.000	1.000	1.000	1.000	**<0.001**
Vehicle vs.	CBD	**0.006**	1.000	**<0.001**	0.124	**0.007**	**<0.001**
Vehicle vs.	THC	1.000	1.000	1.000	1.000	0.330	1.000
Vehicle vs.	BR	1.000	1.000	1.000	1.000	1.000	1.000

One way ANOVA followed by Bonferroni correction (SPSS). $p < 0.05$ were considered significant and are highlighted in bold.

Table S6. Exact p values for the data shown in figure 7.

			HO-1	HO-2	IL6	GRP78	Xbp1	CHOP
§	Vehicle vs.	CBD	**0,045**	**0,025**	**<0.001**	1.000	0.128	**<0.001**
		THC	1.000	1.000	1.000	1.000	1.000	1.000
*	Vehicle vs.	Vehicle + Rot	1.000	1.000	1.000	0.348	1.000	**<0.001**
		Vehicle + BR	1.000	1.000	1.000	1.000	1.000	1.000
		Vehicle + BR + Rot	1.000	1.000	1.000	0.120	1.000	**<0.001**
#	Vehicle + Rot vs.	Vehicle + BR + Rot	1.000	1.000	1.000	1.000	1.000	1.000
*	CBD vs.	CBD + Rot	0.229	0.182	**<0.001**	**0.021**	0.126	**<0.001**
		CBD + BR	0.811	1.000	**<0.001**	1.000	1.000	**0.003**
		CBD +BR + Rot	1.000	0.168	**<0.001**	1.000	1.000	**0.014**
#	CBD + Rot vs.	CBD + BR + Rot	**0.003**	1.000	0.087	**0.037**	1.000	**<0.001**
*	THC vs.	THC + Rot	1.000	1.000	1.000	1.000	1.000	**<0.001**
		THC + BR	1.000	1.000	1.000	1.000	1.000	1.000
		THC + BR + Rot	1.000	1.000	1.000	1.000	1.000	**<0.001**
#	THC + Rot vs.	THC + BR + Rot	1.000	1.000	1.000	1.000	1.000	1.000

One way ANOVA followed by Bonferroni correction (SPSS); signs used to display the significances are given in the first row. p<0.05 were considered significant and are highlighted in bold.

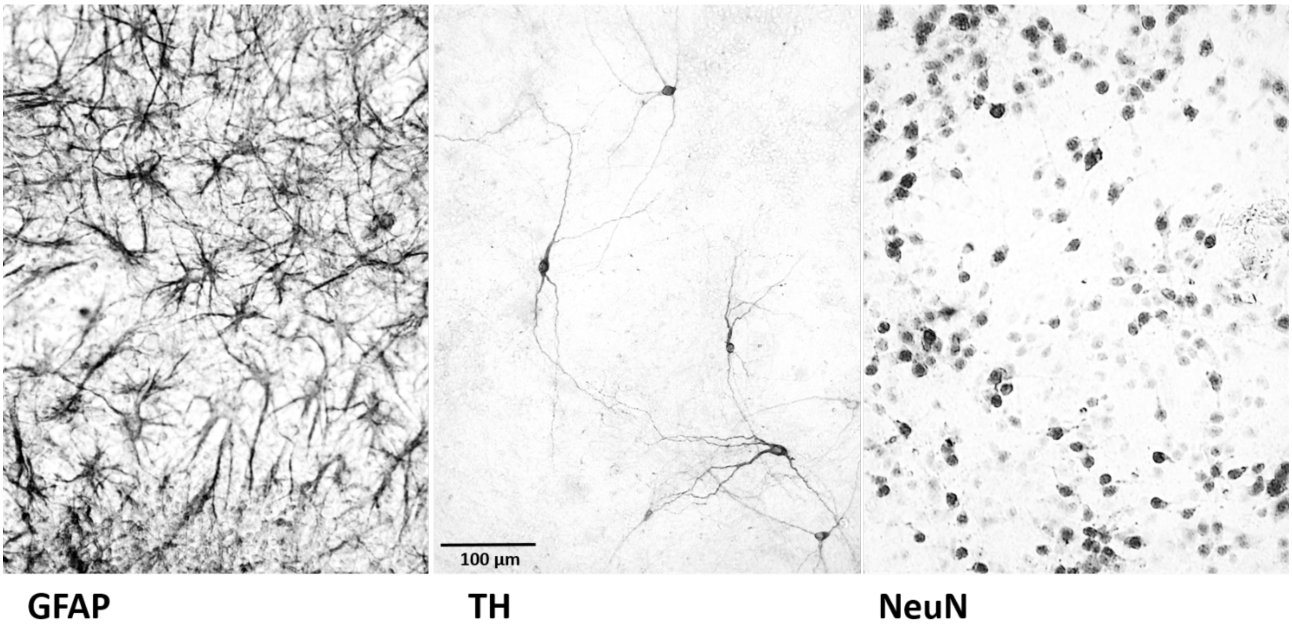

Figure S3. Immunocytostaining against glial fibrillary acidic protein (GFAP; 1:1000, Millipore, Germany), tyrosine hydroxylase (TH) and the neuronal nuclei marker NeuN (1:1000, Millipore, Germany) in mesencephalic cultures. Anti-GFAP and -NeuN staining (both 1:1000, Merck Millipore, Germany) was done according to the method described in 2.1.3. The number of dopaminergic neurons represent less than 1% of the total cell population.

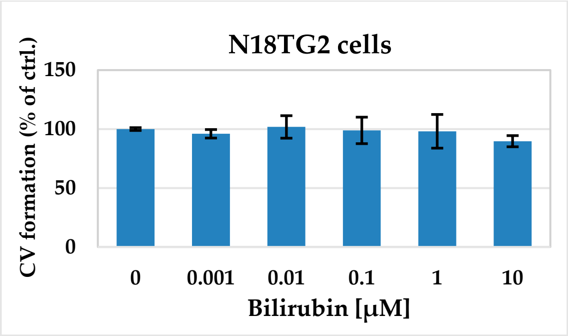

(a)

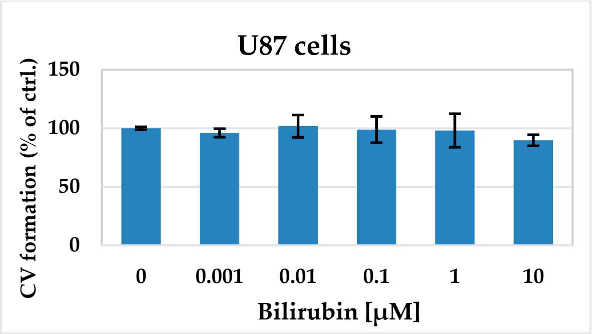

(b)

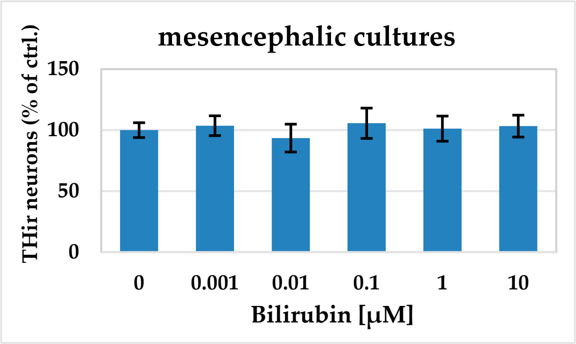

(c)

Figure S4: Effects of bilirubin on N18TG2 and U87 cells or dopaminergic neurons in primary cultures. Cells were treated with different concentrations of bilirubin (BR) for 48 h. Neuroblastoma and astrocytoma cells were stained with crystal violet (**a, b**), and dopaminergic neurons in mesencephalic primary cultures for tyrosine hydroxylase (TH, **c**) before counting. Data are shown as mean +/- SEM of four (a,b) of five (c) independent experiments, respectively. No statistical significance was detected using the Kruskal–Wallis (H) test.

Table S7. Effect of bilirubin on resazurin formation.

**N18TG2 neuroblastoma cells**						
**BR conc. [µM]**	**0**	**0.001**	**0.01**	**0.1**	**1**	**10**
mean	100	99.296	93.338	98.522	94.609	96.621
SEM	1.523	5.578	2.57	2.683	1.972	2.221

**U87 astrocytoma cells**						
**BR conc. [µM]**	**0**	**0.001**	**0.01**	**0.1**	**1**	**10**
mean	100	106.943	107.708	102.986	108.459	108.613
SEM	0.598	6.684	5.686	7.537	7.652	6.162

**mesencephalic primary cells**						
**BR conc. [µM]**	**0**	**0.001**	**0.01**	**0.1**	**1**	**10**
mean	100	99.43	100.238	97.833	97.786	95.361
SEM	2.117	3.012	3.438	2.712	2.828	3.147

In accordance to the crystal violet and tyrosine hydroxylase data, bilirubin does not affect the overall metabolism measured by the formation of resazurin. Data present the mean and the SEM of 10 (neuroblastoma cells) or 4 (astrocytoma cells and mesencephalic cultures) independent experiments, respectively. The Kruskal–Wallis (H)-test followed by the $\chi 2$-test revealed no significant difference between groups.

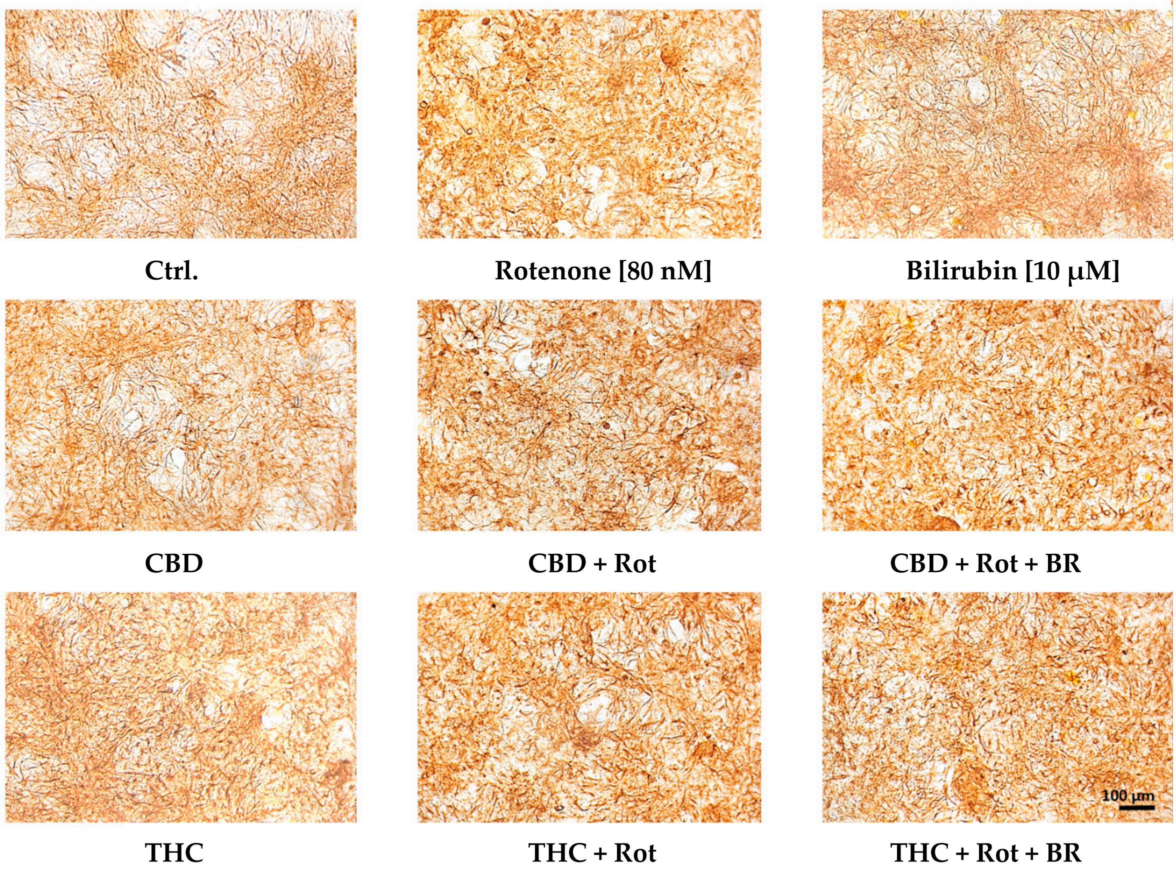

Figure S5. Glial fibrillary acidic protein (GFAP) immunocytochemistry in mesencephalic cultures. Anti-GFAP staining (both 1:1000, Merck Millipore, Germany) was done according to the method described in 2.1.3.

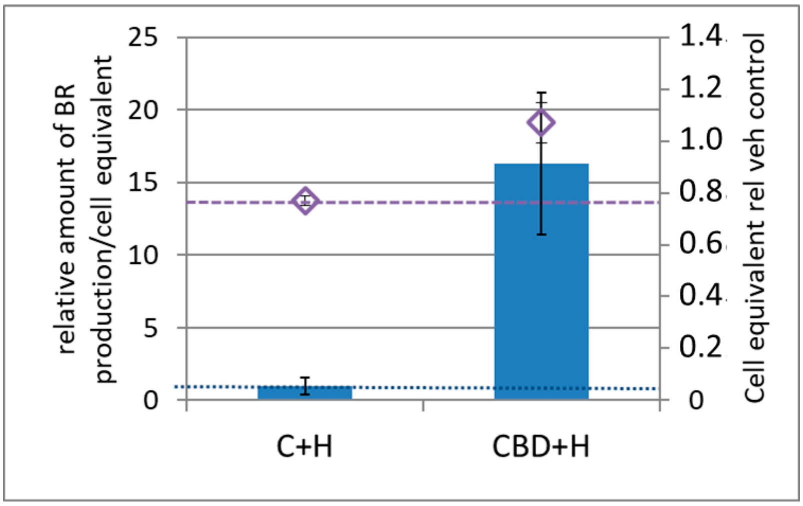

Figure S6. CBD accelerates BR formation from heme by an increased the HO reaction. N18TG2 cells were treated with heme (10µM) with or without adding CBD (10µM). The formed BR was extracted from the cell culture medium after 50 h. 150 µl of medium was supplemented with 50µl caffeine solution and 50µl KCl and the samples treated as the samples obtained in the HO-assay, as described in the Material and Methods section. Amount of BR in benzene-extracts, determined by spectrophotometry, was corrected for the underlying cell number (blue bars, primary Y-axis). Cell number was determined using the crystal violet assay and calculated as cell equivalent (violet open diamond, secondary Y-axis) given relative to the control cells, which were treated by vehicle only and set to 1 (veh control). BR extracted from the control cells was below the detection limit. Data from one experiment performed in triplicates are given as means +/- SD.
